# Switch from enzyme replacement therapy to oral chaperone migalastat for treating fabry disease: real-life data

**DOI:** 10.1038/s41431-020-0677-x

**Published:** 2020-07-09

**Authors:** Eleonora Riccio, Mario Zanfardino, Lucia Ferreri, Ciro Santoro, Sirio Cocozza, Ivana Capuano, Massimo Imbriaco, Sandro Feriozzi, Antonio Pisani, AFFIINITY Group, Antonio Pisani, Eleonora Riccio, Sirio Cocozza, Ciro Santoro, Roberta Esposito, Massimo Imbriaco, Camilla Russo, Teodolinda Di Risi, Lorenzo Chiariotti, Letizia Spinelli, Andrea Pontillo, Alberto Cuocolo, Gilda Cennamo, Annamaria Colao

**Affiliations:** 1 Department of Public Health, Chair of Nephrology, University Federico II of Naples, Via Pansini 5, 80131 Naples, Italy; 2 IRCCS SDN, Naples, Italy; 3 Department of Advanced Biomedical Sciences, Federico II University of Naples, Naples, Italy; 4 Nephrology and Dialysis Department, Belcolle Hospital, Viterbo, Italy; 5 CEINGE-Advanced Biotechnology, s.c.a.r.l, 80145 Naples, Italy; 6 Department of Public Health, Eye Clinic, University Federico II of Naples, Via Pansini 5, 80131 Naples, Italy; 7 Department of Clinical Medicine, Chair of Endocrinology, University Federico II of Naples, Via Pansini 5, 80131 Naples, Italy

**Keywords:** Genetics research, Drug regulation

## Abstract

The treatment options for Fabry disease (FD) are enzyme replacement therapy (ERT) with agalsidase alfa or beta, and the oral pharmacological chaperone migalastat. Since few data are available on the effects of switching from ERT to migalastat, we performed a single-center observational study on seven male Fabry patients (18–66 years) to assess the effects of the switch on renal, cardiac, and neurologic function, health status, pain, lyso-Gb3, α-Gal A activity and adverse effects. Data were retrospectively collected at time of diagnosis of FD (baseline, T0), and after 12 months of ERT (T1), and prospectively after 1 year of therapy with migalastat (T2). No patient died or reported renal, cardiac, or cerebrovascular events during the study period. The predefined measures for cardiac, renal and neurologic function, and FD-related symptoms and questionnaires were stable between baseline and the switch, and remained unchanged with migalastat. However, a significant improvement was observed in left ventricular mass index from baseline to T2 (*p* = 0.016), with a significative difference between the treatments (*p* = 0.028), and in median proteinuria from T2 vs T1 (*p* = 0.048). Moreover, scores of the BPI improved from baseline to T1, and remained stable with migalastat. Plasma lyso-Gb3 levels significantly decreased from baseline to T1 (*P* = 0.007) and T2 (*P* = 0.003), while did not significantly differ between the two treatments. α-Gal A activity increased from T0 to T2 (*p* < 0.0001). The frequency of adverse effects under migalastat and ERT was comparable (28% for both drugs). In conclusion, switching from ERT to migalastat is valid, safe and well tolerated.

## Introduction

Fabry disease (FD) is an X-linked disorder caused by lysosomal α-galactosidase A (α-Gal) deficiency, with subsequent deposition of undegraded glycosphingolipid products, mainly globotriaosylceramide (Gb3) and globotriaosylsphingosine (lyso-Gb3), in multiple organs, with significant morbidity and premature death [1].

Until recently, the treatment options for this genetic disease were limited to enzyme replacement therapy (ERT) with agalsidase alfa or beta. Although long-term data have shown positive effects on disease progression [2–4]; however, ERT is limited by several factors, including considerable clinical variation, high costs, a frequent incidence of mild to moderate infusion-related reactions (which may arise from immunoglobulin antibody formation specific to the infused enzyme), and a life-long burden of biweekly intravenous infusions [5–7].

A new therapeutic approach, represented by the chaperone migalastat, has been commercially available in Europe since 2016. Migalastat is the only oral treatment for FD, which can both be used as a first-line therapy in ERT-naïve patients, and as a suitable alternative to ERT. It reversibly binds to the active site and stabilizes specific mutant forms of α-Gal, defined “amenable” to migalastat, promoving trafficking to lysosomes, where it allows the enzyme to catabolize accumulated substrates [8–11]. It was estimated that 35–50% of Fabry patients have a migalastat-amenable mutation [12].

Although there has not been yet a consensus on when to choose migalastat over ERT, some criteria have been developed, which include: age 16 years and older, a confirmed amenable mutation, an eGFR > 30 mL/min/1.73 m^2^, compliance with every-other-day oral administration, and no intention by female patients to become pregnant. Patients’ preference and hypersensitivity to ERT are also factors in considering the best treatment option [13]. Theoretically, the chaperoning of α-Gal by migalastat to lysosomes may better mimic natural enzyme trafficking and result in more constant α-Gal activity than ERT [12].

To date, different evidences in the literature have confirmed the efficacy and tolerability of migalastat, at the dose of 123 mg every other day, in FD patients with amenable mutations [11, 12, 14]; however, data on the effects of switching from ERT with agalsidase alfa or beta to migalastat are limited to the results coming from the phase III study [12] and the real world study [15].

Therefore, we aimed to assess the effect of switching from ERT with agalsidase alfa or beta to migalastat in real life on renal, cardiac and neurologic function, health status, pain, lyso-Gb3 and adverse events in patients with FD, by comparing retrospective data during ERT with prospective data during treatment with migalastat.

## Methods

### Study population

In this single-center observational study conducted at the University Hospital Federico II of Naples, Italy, a cohort of 7 male patients with genetically confirmed FD and migalastat-amenable mutations, and switched from a stable treatment with agalsidase alfa or beta to migalastat, were consecutively recruited.

Inclusion criteria were the following: (1) adult male patients (≥18 years of age) with genetically determined FD; (2) amenable GLA variant (final determination of amenability was based on the amenability table reported in the SmPc and on the Good Laboratory Practice [GLP]-validated migalastat amenability assay) [16, 17]; (3) patients switched from at least 1 year of stable treatment with the regular dose of agalsidase alfa or beta (0.2 mg/kg and 1.0 mg/kg, respectively, every other week) to migalastat at the approved dose of 123 mg every other day, without any interval; (4) estimated GFR (eGFR) ≥30 mL/min/1.73 m^2^; (5) ≥90% completeness of the mandatory predefined organ and symptomatic data (see below); and (6) informed consent for participation in the study. Subjects changing therapy more often than twice were excluded.

Patients who fulfilled the inclusion criteria gave written informed consent and were followed-up for 12 months; those who have not completed the 12-month follow-up period were excluded from the analysis. The study was conducted in accordance with the Declaration of Helsinki and was approved by the institutional ethic committee.

### Study procedures

In patients identified by the inclusion criteria, data of interest were retrospectively collected at baseline (time of diagnosis of FD, pre-ERT; T0), and after 12 months of ERT with agalsidase alfa or beta (T1), and prospectively after 12 months of therapy with migalastat (T2). A complete clinical assessment was performed in all patients at each visit, including medical history and cardiac, renal, and neurologic evaluation. Use of concomitant medications, including pain medications and antihypertensive agents was recorded. In addition, α-Gal A enzyme activity was assessed in dried blood spots by tandem mass spectrometry at baseline and at the end of observation period (T2).

### Study endpoints

To quantify the clinical outcome, the following groups of FD-related progression parameters were analyzed.

#### Clinical events

These included (i) death; (ii) cardiac events, such as symptomatic arrhythmia requiring implantation of an implantable cardioverter-defibrillator or pacemaker, myocardial infarction, coronary artery bypass graft, or percutaneous transluminal coronary angioplasty; (iii) renal events, such as progression of chronic kidney disease (CKD) to stage 5, i.e., estimated glomerular filtration rate (eGFR) <15 ml/min per 1.73 m^2^ (with decrease of eGFR ≥30%) necessitating kidney transplantation or dialysis; and (iv) cerebrovascular events, such as stroke or transient ischemic attack.

#### Changes in organ function or structure

Modifications of organ function were investigated at the cardiac, renal, and neurologic levels.

Cardiac changes were evaluated by echocardiographic (such as thickness of cardiac structures, left ventricular volume, measures of systolic and diastolic function, heart rate) and electrocardiographic data.

Renal function was evaluated by changes in eGFR, quantified using the CKD–Epidemiology Collaboration Equation [18], and in 24-h proteinuria.

Neurologic changes were determined on the basis of clinical examination, interview regarding stroke or stroke-like symptoms, and evidences of magnetic resonance imaging (MRI).

#### Changes in FD-related symptoms and questionnaires

These symptoms included gastrointestinal pain; diarrhea; hypohidrosis or anhidrosis; tinnitus; acroparesthesia, chronic pain, and pain crises, as assessed by the Brief Pain Inventory questionnaire [19]; fatigue; the Mainz Severity Score Index (MSSI) [20]; and quality of life (QoL), as determined by the Short Form 36 (SF-36) [21], or Euro-Qol dimensions [22].

#### Changes in lyso-Gb3 plasma concentrations

Lyso-Gb3 plasma concentrations (ng/mL) were measured retrospectively at baseline (T0), after 12 months of ERT with agalsidase alfa or beta (T1), and after 12 months of therapy with migalastat (T2).

#### Adverse effects

The adverse effects (AE) considered were dyspnea, hypertension, gastrointestinal symptoms, rigors, temperature change sensation, fever, headache, rhinitis, flushing, pruritus, and antibody formation.

### Statistical analysis

Data are mean ± standard deviation (SD) unless specified otherwise. Since the sample size was less than 30, we used Shapiro-Wilk normality test to first test the normality of the distributions. Subsequently, to compare T0, T1, and T2 data distributions we used paired samples t-test or Wilcoxon-test according to distribution type. In case of normal distribution of data we used paired t-test otherwise we used Wilcoxon signed-rank test. All analyses were performed in R script using basic functions and Paired Data R package (https://cran.r-project.org/web/packages/PairedData/index.html). Value of *p* < 0.05 was considered statistically significant.

## Results

### Demographics and baseline characteristics

Of the 30 male patients with FD receiving ERT at our institution and with amenable mutation, 9 switched to migalastat after the availability of the drug in Italy (30%) and were considered for recruitment; the reasons of the switch are listed in Table 1. Of these, 2 patients had later withdrawn their consent to start therapy, and were excluded from the study; therefore, 7 patients fullfilled our inclusion criteria and were finally enrolled. Patients’ characteristics are shown in Table 1. Moreover, the data of study population have been submitted on GV shared LOVD database (Submitted data are available on https://databases.lovd.nl/shared/individuals/00300304-07). Patients (18–66 years of age) had been on ERT (six on agalsidase alfa, 85.7 %, and one on agalsidase beta, 14.3 %) for an average of 13.3 months. A non-classic (late-onset) GLA variant was found in 2 patients (28.6%; c.1066C>T and c.337T>C) and 5 patients (71.4%) had variants associated with the classical phenotype (c.902G>C and c.901C>G). Fabry-specific laboratory markers showed average lyso-Gb3 plasma levels higher than normal (5.42 ± 4.24 ng/mL; normal values ≤1.8 ng/mL), and a mean enzyme activity of 0.94 ± 0.91 µmol/L/h, that increased at the end of observation period (T2) to 14.42 ± 2.92 µmol/L/h (*p* < 0.0001). Concomitant medications remained unchanged during all the study period.

**Table 1:** Baseline characteristics of enrolled patients.

ID	GLA mutation	α-Gal A activity (µmol/L/h)	Age at baseline (years)	Sex	Previous ERT	ERT duration (months)	Reason for switch
1	c.1066C>T	0.5	66	M	Agalsidase beta	12	AE (nausea, flushing)
2	c.902G>C	3	20	M	Agalsidase alfa	12	AE (fever, (flushing)
3	c.901C>G	0.8	27	M	Agalsidase alfa	12	Patient choice
4	c.902G>C	0.8	22	M	Agalsidase alfa	13	Patient choice
5	c.337T>C	0.5	76	M	Agalsidase alfa	12	Patient choice
6	c.901C>G	0.5	16	M	Agalsidase alfa	20	Patient choice
7	c.901C>G	0.5	38	M	Agalsidase alfa	12	Patient choice

*α-Gal A* α-galactosidase A, *AE* adverse event, *ERT* enzyme replacement therapy, *M* male.

### Clinical events

No patient died and no patient reported renal, cardiac or cerebrovascular events during both ERT and migalastat therapy period.

### Changes in organ function or structure

The predefined measures for cardiac, renal and neurologic function were stable between the retrospective baseline visit and the switch, and remained unchanged during the therapy with migalastat (Table 2).

**Table 2:** Selected study endpoints at baseline (T0), after 12 months’ treatment with ERT (T1) and after 12 months’ treatment with migalastat (T2).

	T0	T1	T2
Renal function			
eGFR (mL/min/1.73 m^2^)	102.57 ± 40.45	99.85 ± 41.11	98.28 ± 40.46
Proteinuria (mg/24 h)	145.00 ± 237.19	135.00 ± 177.69	78.57 ± 128.63^a^
Cardiac parameters			
LVMI (g/m^2^)	39.58 ± 12.30	39.71 ± 9.93	37.17 ± 11.09^a,b^
IVSWT (mm)	10.14 ± 2.47	10.00 ± 2.23	9.71 ± 2.05
LVPWT (mm)	9.00 ± 2.51	9.28 ± 2.21	8.85 ± 2.34
LVEF (%)	59.71 ± 4.38	60.42 ± 4.68	58.42 ± 5.74
E/A	1.36 ± 0.40	1.49 ± 0.45	1.47 ± 0.56
Lyso Gb3 (ng/mL)	5.42 ± 4.24	2.57 ± 1.63^b^	2.12 ± 1.19^b^

Data are expressed as mean ± SD or median (IQR).

*E/A* early to late diastolic transmitral flow velocity, *eGFR* estimated glomerular filtration rate, *IVSWT* interventricular septum wall thickness, *LVEF* ejection fraction, *LVMI* left ventricular mass index, *LVPWT* left ventricular posterior wall thickness.

^a^Significantly different vs T1 (*p* < 0.05).

^b^Significantly different vs baseline (*p* < 0.05).

However, a significant improvement was observed in left ventricular mass index (LVMI) from baseline to T2 (*p* = 0.016), with a significative difference between the two treatments (*p* = 0.028).

Individual changes in cardiac and renal parameters measured at baseline, after the ERT period, and after 12 months of therapy with migalastat are respectively shown in Tables 3 and 4. Table 3 showed that evaluated systolic and diastolic data were generally normal at baseline in the majority of patients and remained stable over both treatment periods. Otherwise, patients with altered cardiac parameters at baseline, did not report significant improvement after both therapies (Table 3).

**Table 3:** Individual echocardiographic cardiac parameters measured at baseline (T0), after 12 months of ERT (T1) and after 12 months of therapy with migalastat (T2).

ID	LVMI (g/m^2^)			IVSWT (mm)			LVPWT (mm)			LVEF (%)			E/A		
	T0	T1	T2	T0	T1	T2	T0	T1	T2	T0	T1	T2	T0	T1	T2
**1**	57	54.3	52.5	14	13	13	13	13	13	56	56	56	0.71	0.71	0.71
**2**	32.2	32.7	32.7	9	10	10	8	8	8	56	65	53	1.88	1.7	1.47
**3**	25	32.1	26	7	8	8	6	8	7	63	63	66	1.27	2.06	1.66
**4**	46	42.4	42.5	10	10	9	8	8	9	60	60	58	1.65	1.62	1.49
**5**	53	52	50	13	13	12	12	12	11	55	55	55	1	1.1	1
**6**	31.5	33.5	26.5	9	8	8	8	8	7	61	57	54	1.59	1.81	2.5
**7**	32.4	31	30	9	8	8	8	8	7	67	67	67	1.46	1.46	1.46

*E/A* early to late diastolic transmitral flow velocity, *IVSWT* interventricular septum wall thickness, *LVEF* ejection fraction, *LVMI* left ventricular mass index, *LVPWT* left ventricular posterior wall thickness.

**Table 4:** Individual renal parameters and lyso-Gb3 plasma levels measured at baseline (T0), after 12 months of ERT (T1) and after 12 months of therapy with migalastat (T2).

ID	eGFR (mL/min/1.73 m^2^)			Proteinuria (mg/24 h)			Lyso-Gb3 (ng/mL)		
	T0	T1	T2	T0	T1	T2	T0	T1	T2
**1**	36	36	36	665	500	350	7	3.2	3
**2**	131	130	130	150	150	0	0.8	0.5	0.5
**3**	123	122	122	0	0	0	3.2	2.3	2.2
**4**	128	121	133	100	170	100	1.2	0.5	0.6
**5**	53	45	45	0	0	0	12.5	3	2
**6**	133	132	109	0	125	100	4.6	3.5	3.5
**7**	114	113	113	100	0	0	8.7	5	3.1

*eGFR* estimated glomerular filtration rate.

Similarly, individual eGFR values showed that at baseline eGFR was normal (eGFR ≥ 90 mL/min/1.73 m^2^) in 5 of the 7 patients, and remained normal during both the 12-month periods of ERT and of migalastat. Two patients had low eGFR levels at the baseline, that remained unchanged at the time of switch of therapy, and did not worsen under both treatments (Table 4). Moreover, median proteinuria showed a significative decrease in T2 vs T1 (*p* = 0.048), and individual patient data showed that only one patient had relatively high proteinuria at baseline (pt 1), that showed a small, not significant improvement with both drugs (Table 4).

### Changes in FD-related symptoms and questionnaires

Most of predefined measures remained stable during both treatment periods. Only scores of the BPI improved from baseline during treatment with ERT, and then remained stable during treatment with migalastat (data not shown). In particular, of the 7 patients, 5 had no pain at baseline and remained pain free during all the follow-up period; while the 2 patients with pain at baseline reported an improvement of the pain during ERT, and a further amelioration during the therapy with migalastat.

### Changes in lyso-Gb3 plasma concentrations

Plasma lyso-Gb3 levels showed a significant reduction from baseline under ERT (*P* = 0.007), and under migalastat (*P* = 0.003) (Table 2). Moreover, plasma lyso-Gb3 levels did not significantly differ between the two treatments. Individual changes of lyso-Gb3 concentrations measured at baseline, after 12 months of ERT and after 12 months of therapy with migalastat are shown in Table 4.

### Adverse effects

Treatment with migalastat was generally safe and well tolerated. The frequency of treatment-emergent AE under migalastat and ERT was comparable (2 patients—28%—for both drugs).

In particular, the AE observed in two patients during the ERT period resolved with the switch to migalastat; conversely, 2 patients respectively reported rynhitis and headache during treatment with migalastat, not observed under ERT. Most AE were mild in severity. No patient discontinued study drug due to a treatment-emergent AE.

## Discussion

As an orally administered small-molecule agent, migalastat may obviate the burden of life-long administration of ERT every 2 weeks. Theoretically, chaperoning misfolded α-galactosidase to lysosomes may better mimic natural enzyme trafficking and result in more consistent α-gal activity than enzyme-replacement infusions every 2 weeks. Although the pharmacological kinetics of the migalastat and its reversible bond with the α-galactosidase in vivo should be taken into consideration when we refer to the biological activity of migalastat [23]. Moreover, migalastat would avoid ERT-associated immunogenicity and infusion-associated reactions; in fact, in a 5-year retrospective analysis in patients treated with ERT, 40% of males had serum-mediated antibody inhibition of agalsidase activity, which was associated with higher lyso-Gb3, greater left ventricular (LV) mass and decreased renal function [24]. Additionally, the higher volume of distribution of migalastat (76.5–133 L) [25] relative to ERT suggests enhanced penetration of organs and tissues [26].

The efficacy of oral migalastat at the approved dose of 123 mg every other day, in patients aged 16–74 years with genetically confirmed FD, was assessed in two pivotal, randomized, multicentre, placebo-controlled (FACETS [11]) or active comparator-controlled (ATTRACT [12]) phase 3 trials and long-term open-label extension studies [27, 28].

In particular, results of FACETS study showed that migalastat led to reduced substrates in kidney and plasma, stabilized renal function, reduced cardiac mass, and improved gastrointestinal symptoms in Fabry patients who were either ERT-naïve or had not received ERT within the past 6 months [11]. A following paper by Germain et al [29], aimed to assess the clinical benefit of migalastat in the subset of male patients with the classic phenotype in the FACETS trial, provided additional evidence for its beneficial effects for up to 24 months.

More interestingly, the phase 3, active-controlled ATTRACT trial assessed the effects of migalastat in Fabry patients previously treated by ERT. The authors showed that renal function was maintained during 18 months of migalastat or ERT; moreover, migalastat significantly reduced cardiac mass compared with ERT, and no difference was reported on the other evaluated endpoints (renal, cardiac or cerebrovascular events, plasma lyso-Gb3 concentration, and patient-reported outcomes) [12]. Finally, the switch of treatment was well tolerated, as highlighted by the same authors in the following paper [13].

Moreover, the efficacy of migalastat in the real-world setting has been evaluated in a prospective single-center study in patients with migalastat-amenable mutations of α-galactosidase A [15], that showed that migalastat therapy led to a rapid, persistent increase in α-galactosidase A activity in both male and female amenable Fabry patients. Over a follow-up period of 12 months, higher enzyme activity was associated with a trend in reduced lyso-Gb3 levels and a significant reduction in cardiac hypertrophy. Interestingly, data of subgroup of the 6 patients switched to migalastat from a previous ERT, confirmed that both treatments had comparable effects on all evaluated endpoints, with an improvement only of α-galactosidase A activity after switching.

Our study confirms these results, although the short follow-up period of therapy with the migalastat. We reported that renal, cardiac and neurologic function, pain symptoms and health status were unchanged in our population of 7 male Fabry patients with amenable mutations switched from 12 months of ERT with agalsidase alfa or beta to migalastat for 1 year, with the exception of a small but significant amelioration in LVMI (−2.41 g/m^2^) and proteinuria with migalastat vs ERT.

Moreover, therapy with migalastat led to an increase in α-galactosidase A activity and reduction in lyso-Gb3 levels, suggesting that patients maintained disease stability. Finally, the switch of treatment was well tolerated.

The present study has some important limitations: it was conducted at a single center, the cohort only comprised 7 patients and the follow-up period is short. These factors limit the statistical power of our results, which must be considered when judging the validity of all statistical analyses. Therefore, the present findings should be confirmed in the future with multicenter studies that use longer observation periods.

In conclusion, in Fabry patients with amenable mutations, switching from ERT with agalsidase alfa or beta to the pharmacological chaperone migalastat seems to be valid, safe and well tolerated.
